# For the love of fish, nature and people: A tribute to Professor Louis Bernatchez (1960–2023)

**DOI:** 10.1111/eva.13609

**Published:** 2023-11-16

**Authors:** Maren Wellenreuther, Anne‐Laure Ferchaud, Luciano B. Beheregaray, Nicolas Bierne, Alberto Soares Correa, Kathryn Elmer, Dylan Fraser, Danna Gifford, Zachariah Gompert, Marc Johnson, Rees Kassen, Ryan Martin, Mariah Meek, Joachim Mergeay, Claire Mérot, Kerry Naish, Shawn Narum, Paul Sunnucks, Frédéric Thomas, Peter Thrall, José Manuel Yáñez, Jiasui Zhan, Xiangjiang Zhan

**Affiliations:** ^1^ The New Zealand Institute for Plant and Food Research Limited Auckland New Zealand; ^2^ University of Auckland Auckland New Zealand; ^3^ Parks Canada Agency within the Ecosystem Science Laboratory of the Office of the Chief Ecosystem Scientist Québec City Quebec Canada; ^4^ Flinders University Adelaide South Australia Australia; ^5^ CNRS at Institute of Evolutionary Science of Montpellier, University of Montpellier Montpellier France; ^6^ Department of Entomology and Acarology at the University of São Paulo Piracicaba São Paulo Brazil; ^7^ School of Biodiversity, One Health & Veterinary Medicine University of Glasgow Glasgow UK; ^8^ Department of Biology Concordia University Montreal Quebec Canada; ^9^ School of Biological Sciences University of Manchester Manchester UK; ^10^ Department of Biology Utah State University Logan Utah USA; ^11^ University of Toronto Toronto Ontario Canada; ^12^ McGill University & University of Ottawa Ottawa Ontario Canada; ^13^ Department of Biology Case Western Reserve University Cleveland Ohio USA; ^14^ Department of Integrative Biology Michigan State University Ann Arbor Michigan USA; ^15^ Katholieke Universiteit Leuven & Research Institute for Nature and Forest Leuven Belgium; ^16^ CNRS at institute ECOBIO University of Rennes Rennes France; ^17^ School of Aquatic and Fishery Sciences University of Washington Seattle Washington USA; ^18^ Fishery Science Department, Hagerman Genetics Lab Columbia River Inter‐Tribal Fish Commission Portland Oregon USA; ^19^ School of Biological Sciences Monash University Melbourne Victoria Australia; ^20^ Centre for Ecological and Evolutionary Research on Cancer (CREEC), and UMR CNRS (Maladies Infectieuses et Vecteurs: Ecologie, Génétique, Evolution et Contrôle) Montpellier France; ^21^ Commonwealth Scientific & Industrial Research Organisation (CSIRO) Canberra New South Wales Australia; ^22^ Facultad de Ciencias Veterinarias y Pecuarias Universidad de Chile Santiago Chile; ^23^ Swedish University of Agricultural Sciences Uppsala Sweden; ^24^ Key Laboratory of Animal Ecology and Conservation Biology, Institute of Zoology Chinese Academy of Sciences Beijing China

It is difficult to overstate the profound impact that Professor Louis Bernatchez had on the wider field of genomics applied to the conservation and management of aquatic resources. Louis died on 28 September 2023 at the age of 63. His passing represents an immense loss. He has held the position of Editor‐in‐Chief for *Evolutionary Applications* since its inception in 2008, and his unwavering passion and commitment to making science relevant for a wide range of applications and end‐users have significantly influenced the journal's trajectory. We remember him as an exceptionally compassionate, encouraging and genuine individual, possessing unparalleled scientific expertise, and boundless generosity (Figure 1). Our hearts go out to his family, friends, colleagues and students during this incredibly challenging period.

**FIGURE 1:**
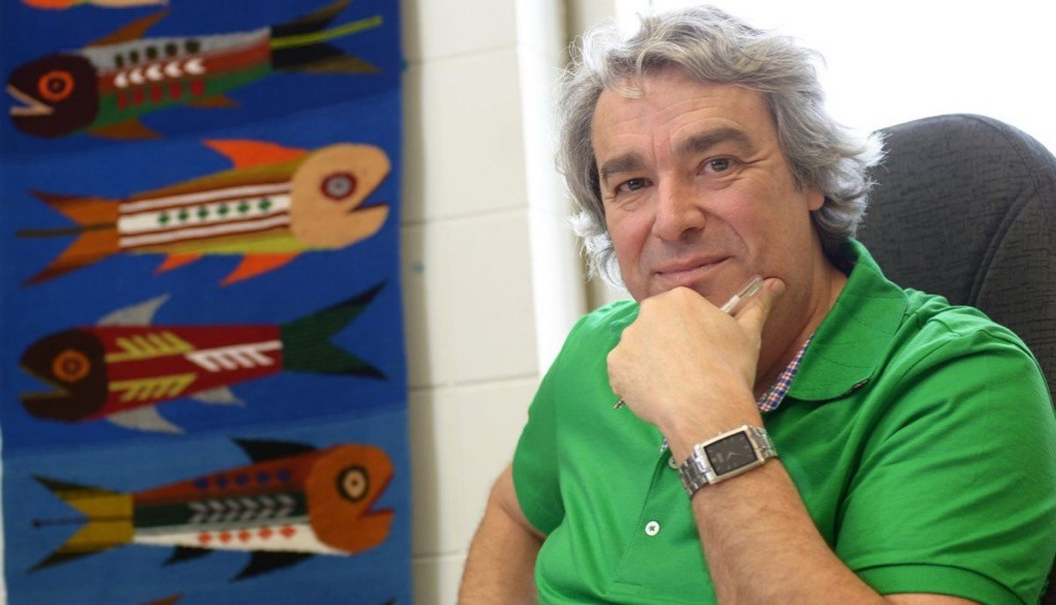
Louis Bernatchez in his office in 2018 at Laval University, Québec, Canada. Photo taken by Marc Robitaille.

Born in 1960, Louis grew up in the small and tight‐knit community of Lac‐Frontière in Québec, Canada (Ferchaud et al., 2020). His formative years were spent in the wilderness, engaging in hunting and fishing, which helped to forge a profound and enduring bond with the natural world—a connection that would remain with him throughout his life. At the age of 12, he already harboured a firm resolve to pursue a career as a biologist. In 1983, he earned his BSc from the Université Laval in Québec, Canada. Shortly after in 1986, he completed his MSc degree, researching the energetic costs in Lake Whitefish and Cisco. This research ignited a lifelong passion, leading to 40 years dedicated to exploring microevolution's role in macroevolution using sympatric Lake Whitefish species pairs. Afterwards, he briefly left academia to launch a commercial whitefish harvesting project in the remote Quebec community, Laniel. He returned to academia and earned his PhD in 1990, initially focused on the population genetic structure of coregonine fishes which he expanded into a thesis on large‐scale phylogeography. Following his PhD, he held postdoctoral positions in France (Université de Montpellier II) and Canada (University of Guelph, Ontario) but felt a strong pull to return to Québec. In 1992, he became a Research Associate at the Université du Québec and later an Assistant Professor at the Institut National de la Recherche Scientifique (INRS). Finally, in 1995, he re‐joined the Université Laval, where he rose to full professorship in 2004, took the head of the Institute of Integrative Biology and Systems in 2018, and continued his academic journey until his passing.

Louis had a prolific and productive career spanning four decades. He has supported and co‐authored well over 500 publications and worked with over 100 collaborators (Figure 2). As anyone who worked with Louis knows, most of these collaborators probably enjoyed (at least) a meal or drink with him, over which he passionately discussed politics, science and fishes. This attitude was just a reflection of his restless working style. For him, being a scientist was his way of living, and he combined this with all the other pleasures he had in life, spanning food, politics, wine, time with loved ones and music. Family played a pivotal role in every facet of Louis's life, and his seamless integration of family and his scientific pursuits served as a powerful example of how one could genuinely embrace and celebrate the concept of work–life balance.

**FIGURE 2:**
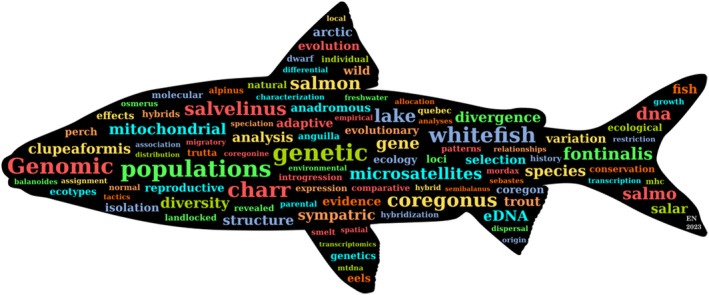
Image created based on Louis' publications from 2002 to 2023 by Eric Normandeau.

As part of his tireless passion for science, he contributed immensely to the growth of new disciplines in the field. He was a pioneer in phylogeography and in the application of functional genomics to non‐model organisms. He stressed the importance of integrating molecular genetics and ecology in understanding adaptive divergence and speciation, and he also championed population genomics, advocating for the holistic integration of genomic, transcriptomic and epigenomic data with ecological, physiological and life‐history data in adaptation studies. He also demonstrated the importance of conservationists engaging with researchers in other disciplines, such as climate science, economics and social science. While he primarily focused on fishes, he expanded his research to include a diverse range of organisms, from invertebrates and birds to mammals. His name is now synonymous with the concepts of ecotype divergence, conservation genomics, genetic health, natural resources management and evolutionary adaptive potential, and his achievements have left a permanent mark on these areas of study.

Louis was highly active in fish research with Indigenous communities across Canada and had a particular soft spot for Eeyou Istchee where he conducted some of his earliest research on whitefish along the James Bay coast. Most recently, he became the Principal Scientist leading the Genome Canada grant *Fostering Indigenous Small‐scale fisheries for Health, Economy and Food Security* (FISHES, https://fishes‐project.ibis.ulaval.ca/about‐fishes/). This FISHES project is a multi‐year project weaving genomics, fisheries science and indigenous knowledge across northern Canada to address critical socio‐economic challenges and opportunities related to food security and commercial, recreational and subsistence fisheries, and Louis was passionate about it.

Louis has received numerous awards, and scientific accolades for his contributions to the scientific advancements of these fields (Ferchaud et al., 2020). Most notable are the Canadian Research Chair in Conservation Genetics of Aquatic Resources and then in Genomics and Conservation of Aquatic Resources, which he held since 2001 and 2006, respectively, and election to the Royal Society of Canada (2011), American Association for the Advancement of Science (2011), receiving the Marie‐Victorin Prix du Québec (2012), the Molecular Ecology Prize (2016) and being elected to become knight of the Ordre National du Québec (2020). Beyond the formal prizes and awards, Louis was humbly delighted that such recognition gave him the opportunity to share science, to communicate broadly about the research of his team and to diffuse important messages for the conservation of biodiversity.

The scientific community will remember him not only for these awards but also for his passion for science, energetic personality and collaborative philosophy, which he incorporated into a unique way of doing research. Those who had the honour and pleasure of knowing him knew that Louis was not only a living scientific hub but also a colleague, teacher, supervisor and mentor who influenced the lives of so many of us in profound ways. He was immensely passionate about raising the next generation of scientists from undergraduate to post‐doctoral researchers. His team, his ‘gang’ as he would have said in québecois, was his highest priority throughout his career including during the last months fighting for his life. This supervising role was his biggest pride, not merely from a scientific point of view, but also as a mentor, who took great care of the future and happiness of his students who were about to take the next step in their careers. Such attention to others was expansive in time—membership of the Bernatchez group had no expiration date—and also in space: Louis had many enduring relationships with collaborators around the world.

Despite the untimely departure of Louis, he would have wanted us to continue treading the path he so brilliantly charted. He would have enjoyed seeing us coming together not just as colleagues but as friends, sharing a glass of wine or a beer, listening to harmonious melodies and revelling in joyful laughter. As we navigate the journey he began, we pay tribute to his enduring spirit and the indelible mark he left upon our lives.

In Box 1, we offer some reflections on Louis' extraordinary journey as Editor‐in‐Chief of *Evolutionary Applications*, and past and present Associate Editors and Guest Editors of the journal share some of their personal thoughts and memories about Louis as leader, storyteller, colleague and mentor. Over the course of his period as Editor‐in‐Chief of *Evolutionary Applications*, Louis has managed a substantial portfolio, encompassing more than 6000 manuscripts.

BOX 1 **Personal reflections of past and present Associate Editors and Special Issue Guest Editors of Evolutionary Applications**

**Luciano Beheregaray, Flinders University, Australia**

It was late September 2000. I had arrived in Brisbane the night before and was as excited as I was frightened about presenting my ongoing PhD work in front of Louis Bernatchez, the world‐famous fish phylogeographer. I was there because of an invitation from Craig Moritz: ‘You should come and visit our lab. Louis is doing a sabbatical with us'. I will be always thankful for that invite. Louis' kindness and humour made me feel immediately at ease. My talk *Biogeography and speciation of South American silverside fishe*s was followed by Louis' brilliant talk *Fish stories from the North: relevance for speciation*. Too many fishes in just 1 day for a herpetology lab! Louis' assertiveness assured me I was on the right track and his huge interest in fish evolution later sparked our long‐term research collaboration across the equator. He acted as my PhD examiner, and years later became the host for my first academic sabbatical, a mentor, a key team member of our rainbowfish projects and field expeditions, a host for my PhD students and postdocs, an inspiring and tireless Editor‐in‐Chief, and an amusing friend. We had the chance to celebrate work and life multiple times, and what always amazed me in those moments was how generous with his time and kind Louis was with students and colleagues (including competitors), as well as with random folks who crossed his path. In this article, we pay tribute to his life, to his immense integrative vision and to his prominent contributions to the fields of ecological, evolutionary and conservation genomics. For me, I will always remember and celebrate Louis as the friendliest and greatest ‘fish guy’ I've ever met.

**Nicolas Bierne, CNRS at Institute of Evolutionary Science of Montpellier, University of Montpellier, France**

I have only met Louis in person three short times, three memorable times though. I have never collaborated with him on a project or an article, and yet today I feel a great emptiness, as great as the loss of a close colleague, or even a member of my family. Louis was like that. He made us feel part of a big family, the molecular ecology family, of which he was a figurehead. And even more so, for the community of aquatic geneticists, that he loved, protected, encouraged and pushed to be the best it could be. For example, he pushed me hard to write one of my rare articles that had little impact in our field, the coupling hypothesis, arguing that discussions over beers at conferences were pointless and that you had to have the courage to act on your convictions. I wish he was still around to keep pushing us that way, opening up the pages of Evolutionary Applications, or other journals he worked tirelessly on, to recent developments, new or alternative ideas. I also shared with him the same language, French, which he defended as a good Quebecer. He was keen to maintain a link with France, and many of my master's and PhD friends passed through Louis' lab for their PhD or post‐doc. Then, the students in my team also wanted to apply to Louis' lab, which was as renowned for the quality of its science as it was for the friendly atmosphere that reigned there. We also owe it to him that the Bernatchez school of thinking and working has spread back throughout France. When he invited me to be an Associate Editor of *Evolutionary Applications* he had instant confidence in me, and this in turn gave me confidence in myself. For 2 years, he put me in charge of inviting reviews and synthesis articles. Once again, he pushed me to hunt for new ideas in the field and supported and stimulated me with his formidable efficiency. We were able to work serenely under the benevolence of the best Editor‐in‐Chief one could hope for. I will miss him so much.

**Alberto Soares Correa, Department of Entomology and Acarology at the University of São Paulo, Brazil**

I was extended an invitation by Louis to join as an Associate Editor. Regrettably, I never had the chance to meet him in person. It deeply saddens me to learn of his passing, and I would like to extend my heartfelt condolences to his family and friends.

During my brief time contributing to *Evolutionary Applications*, I have observed a significant interest among the editorial team in the application of evolutionary biology in the field of agriculture. I believe that this is a remarkable initiative, particularly considering that other journals in this area do not share the same enthusiasm. From the message I received from Louis inviting me, it appears that he was immensely passionate about the Journal and to show the importance of understanding the evolutionary processes in the agricultural landscape, where the evolutive processes happen fast and are strongly influenced, though not entirely controlled, by human activities.

**Kathryn Elmer, School of Biodiversity, University of Glasgow, UK**

Louis was such a positive and pioneering scientist, it was really wonderful to discuss with him. I found him always very supportive and enthusiastic, while also having a pragmatic eye to the right strategy to get things done successfully. He seemed to know and remember everyone! He could always find a bit of time to bat around an idea, offer some advice or return a quick cheery reply … usually closed with ‘enjoy it!’. He was so influential for our community and we will really keenly feel the loss.

**Anne‐Laure Ferchaud, Parks Canada Agency within the Ecosystem Science Laboratory of the Office of the Chief Ecosystem Scientist, Canada**

Louis' conviction and passion for applied biology have positioned him at the very top as a political researcher as well as a fundamental researcher. Always ahead of his time, ready to give visibility to emerging fields or even minorities, he has initiated a very diverse panel of Special Issues in the journal (evolutionary conservation, biodiversity monitoring, fisheries management, forest genomics, agricultural and aquaculture systems, human‐induced evolution, evolution of cancer, evolutionary medicine, bioinvasions, infectious diseases, toxicology, resurrection ecology, epigenetics, adaptation and maladaptation). He mentored hundreds of graduate students, post‐docs, research professionals and research assistants over the years and probably thousands more indirectly via his journals, publications and communications. Louis inspired so many people wherever he went. We will not be grateful enough for his outstanding contributions to the field, invaluable mentorship and boundless generosity and compassion. He will be missed dearly.

**Dylan Fraser, Department of Biology, Concordia University, Canada**

I am fortunate to have known Louis for over twenty years as his PhD student first, as a close research collaborator thereafter, and as an Associate Editor of Evolutionary Applications some time ago. Louis represented a unique combination of alleles: a pioneer and star researcher over many years, but also a very approachable and down‐to‐Earth person. Louis was a magnanimous spirit who tirelessly supported those he mentored and who provided a wealth of tremendous opportunities to others. He inspired many early career researchers as well as established scientists, and had an intangible ability to bring out the best in each individual’s research abilities and creativity, no matter their background. If Louis invited you to join a project or editorial board, you knew you were in for an adventure that would be challenging and a lot of hard work, but also a highly rewarding experience, with some fishing and good food thrown in the mix.

When Louis was encouraging me to join his lab as a graduate student, he left a message on my answering machine describing the proposed project working with trout and Cree First Nations in northern Canada. His message finished with ‘I think we’ll have a lot of fun together’, and he sure was right. I couldn’t have asked for a better academic father in research life, and I am forever grateful for everything Louis taught and instilled in me over the years. If there is a case to be made for designating the Evolutionary Significant Unit to the level of an individual, Louis is surely it.

**Danna Gifford, School of Biological Sciences, University of Manchester, UK**

I first met Louis early in my career at a conference during my master's studies. *Evolutionary Applications* had then recently launched as a journal, and I vividly remember his excitement and enthusiasm for its future. Over a decade later, I was honoured when Louis invited me to join the journal's editorial team. Though our paths never crossed in person again, I am grateful for the advice and feedback he generously provided during my first days as an Associate Editor. Louis made a lasting mark on the ecology and evolution research community in Canada, leaving an enduring legacy that will undoubtedly shape the future for years to come.

**Zachariah Gompert, Department of Biology, Utah State University, USA**

Louis' contributions to the journal and field were, of course, gargantuan. But I would like to highlight his contributions from a personal angle. I first met Louis after giving a talk while I was still a graduate student. He found me to chat afterwards and I was rather moved by the level of respect he conveyed given how junior I was. A few years later, he invited me to be an Associate Editor for *Evolutionary Applications*. I was repeatedly grateful for the way he treated and encouraged me in my early days as an Associate Editor. My experiences are far from unique. Thus, I think the wonderful and encouraging way Louis interacted with up and coming generations of scientists was among his greatest contributions.

**Marc Johnson, University of Toronto, Canada**

In addition to Louis’ role as an outstanding scientist and trailblazing editor of the first journal in applied evolutionary biology, in 2022 he helped to ignite and lead a movement in Canada. Along with Sally Otto and myself, Louis cofounded Support Our Science, the grassroots movement advocating for increased funding for graduate students and postdoctoral scholars. From day 1, he helped to formulate our strategy, attend meetings with government officials, spoke at a large rally in Montreal in front of Prime Minister Trudeau’s office, routinely spoke to the French and English media, and served on our board of directors. As recently as the week before his death he attended our board meeting and was upbeat and contributing without reservation. He was among Canada’s greatest champions fighting for graduate students and postdoctoral scholars.

**Rees Kassen, McGill University and University of Ottawa, Canada**

Two things stand out to me in terms of Louis' contributions to the journal. The first was that he actually managed to get it off the ground and guided it to become an influential venue for publication in the field. So many evolutionary biologists would talk about the potential practical relevance of their research but, before *Evolutionary Applications*, there really was no good place to put it. The major journals in evolutionary science seemed to be more concerned, often—and occasionally unapologetically—on foundational problems in the field. More practical‐focused venues, especially those in ecology and health sciences, often discounted the importance of evolution in their work (or simply called it something else, like competition). *Evolutionary Applications* filled an important gap in the research landscape and, in doing so, normalized and in many ways professionalized the use of evolutionary principles for practical ends.

The second was Louis as a person—he was always generous to me, as I am sure he was with everyone, with his time. Despite being quite senior to me in the Canadian academic world, he never held himself in too high regard in our interactions, always treating me with respect and as a colleague, despite our different ages. I valued that, especially as a young scientist trying to find my way in research. He made me feel I had a place in the research conversation of this country. For that, I am grateful.

He was a warm, generous person and made outstanding contributions to evolutionary science. He will be missed.

**Ryan Martin, Case Western Reserve University, Department of Biology, USA**

I did not know Louis personally outside of the journal but I very much appreciated the wide, yet distinct voice he gave to the journal, threading the needle to highlight the applied research in evolution while keeping that definition inclusive. In doing so, he helped make *Evolutionary Applications* a great home for research in my own pretty young field of Urban Evolution, for which I will always be grateful.

**Mariah Meek, Department of Integrative Biology, Michigan State University, USA**

Louis has been a beacon of inspiration for me throughout my career. As a graduate student, he had almost mythical status in my mind as one of the people defining the field to which I was dedicating myself. And then I got to meet him when I was a post‐doc, and his status in my eyes rose even higher. It is not that often you get to meet one of your heroes and they are so gracious and encouraging. As many others here have said, Louis' encouragement of early career researchers made a mark on so many of us. Louis and his lab continued to light the way as I moved on to start my own lab and I got to interact with him more. I used to joke to people in my lab that every time I had what I was sure was the best idea ever, all I had to do was wait a week and Louis was surely to be publishing a paper on it, doing it much better than I could have dreamed of. We will dearly miss that light he showed for us and do our best to honour him by continuing to carry it forward.

**Joachim Mergeay, Katholieke Universiteit Leuven and Research Institute for Nature and Forest, Belgium**

Louis was a warm, inclusive and inspiring scientist, academic and colleague. In 2011, he contacted me to edit a special issue for *Evolutionary Applications* to bundle the findings of an online conference I had co‐organized. I had just started as a PI, and Louis saw some potential. This turned out to be a career‐changing moment for me. In a contagious way, he oozed pleasure in his work and embodied a passion for the wonder of nature and evolution. Somehow, I always felt welcome in his presence. We shared a life‐long love for fishing, and I will continue to remember and honour his generosity, especially when I am standing in a river casting a fly to lure the next trout.

**Claire Mérot, CNRS at institute ECOBIO, University of Rennes, France**

Louis' attachment to scientific journals originated from a strong feeling that science should be shared. When he asked me to act as a ‘social media editor’ to post about the articles published in *Evolutionary Applications* on social networks, I was really surprised. Tweeting scientific papers may appear, in a way, as unnecessary advertisement on commercial platforms, which does not really fit our ethics and values. However, Louis' idea was very different and changed my feelings about it. His deep motivation was to share scientific results broadly, increase the accessibility of papers, and communicate to a mixed audience of scientists, end‐users and the general public. This accessibility was particularly relevant for studies with concrete applications like the ones published in Evolutionary Applications. Louis' involvement in scientific communication was also reflected by his frequent contributions to local newspapers and audiovisual programs. Many times, did we laugh about cameras coming to Louis' lab to film someone pipetting coloured water! Yet, Louis took all possible opportunities to give back science to citizens. His engagement and his love for talking about science were instrumental in raising awareness about the importance of fundamental research and the conservation of biodiversity, in Québec and at an international level. I believe that Louis' commitment to disseminating science is an important part of his legacy that will keep inspiring many of us.

**Kerry Naish, School of Aquatic and Fishery Sciences, University of Washington, USA**

Louis was key to defining the field of Molecular Ecology—and then, he invited everyone in. His was a very large, rambunctious and creative tent. He paid particular attention to promoting the careers of others, and I found myself swept in with generosity and warmth. I cannot remember when I first met Louis, but I distinctly remember the call to join the *Evolutionary Applications* community—a poor connection between long‐distance flights—which led to many expansive discussions arising from the creative manuscripts that flew by me almost on a daily basis. Our paths crossed often both formally and informally, and I was always stuck with Louis' far‐sightedness, his inventive approach to science, his ability to push boundaries, and always, the way that he treated science as a team game. I count myself very fortunate to have worked with Louis—it has always been a huge amount of fun.

**Shawn Narum, Fishery Science Department, Hagerman Genetics Lab, Columbia River Inter‐Tribal Fish Commission, USA**

Louis had an incredible vision for advancing science that would help shape multiple fields for decades, including evolutionary, ecological, and conservation genomics. He revealed cutting‐edge applications not through only his own studies but also by guiding and encouraging others. Louis was often at the forefront of new genomics technologies, and his insight has ushered in a new era that continues to revolutionize our understanding of the genetic variation of natural species. With the loss of Louis, he passes this incredibly bright torch to the rest of us to carry forward in this world. I am grateful to have known him and experienced his generosity.

**Paul Sunnucks, School of Biological Sciences, Monash University, Melbourne, Australia**

Louis was a giant in molecular population biology, leading and inspiring a mountain of innovative and creative science that contributed hugely to the rapid development of the discipline. This is readily demonstrated by a glance at eye‐watering publication statistics and a very long list of prestigious awards. Louis' energy and work‐rate towards building the field was also extraordinary, including more than two decades as a lead editor of *Molecular Ecology* from close to the start of that foundational journal, and subsequently being the driving force behind *Evolutionary Applications* since its inception. But these achievements do not capture the very special contributions that Louis made to people in the field, through his strengths as an exceptional human being. Some of those influences have been warmly reflected by people who knew Louis well (Ferchaud et al., 2020). However, Louis was also an outstanding role model, touching the professional lives of many, whose debt to him he probably did not realize. As a new Associate Editor of *Molecular Ecology* in the early 2000s, I benefited from invaluable support and encouragement from Louis, long before I met him in person. On several occasions as an author of papers in *Molecular Ecology* and *Evolutionary Applications*, I had such great experiences with Louis's common sense, personality and reasonableness as an editor that I have tried to bring his humorous and human approach into my own editorial handling. Within my own research group and elsewhere I have frequently used Louis and *Evolutionary Applications* as an example of how scientific publishing should be done.

**Frédéric Thomas, Centre for Ecological and Evolutionary Research on Cancer (CREEC), and UMR CNRS (Maladies Infectieuses et Vecteurs: Ecologie, Génétique, Evolution et Contrôle), Montpellier, France**

Twenty‐five years ago, I had the privilege of meeting Louis in Quebec City. From our first meeting, I was immediately captivated by his research and the charisma with which he spoke about it. Louis became a friend with whom I interacted right to the end. His courage was exemplary, even when he realised that he had reached an impasse. His unshakeable determination to demonstrate that evolutionary biology was not just an elegant way of explaining the past, but that it had applications in many fields, contributed greatly to the advancement of our discipline. Today, we are proud to continue on Louis' path, ensuring that his legacy lives on.

**Peter Thrall, Editor in Chief Ecology Letters, and the Commonwealth Scientific and Industrial Research Organisation (CSIRO), Australia**

I worked with Louis for many years on the journal *Evolutionary Applications*, from the very beginning in fact. While I do not believe that Louis and I ever met in person, we corresponded regularly about various topics related to the journal and the field. It was always a pleasure, not just because of the high level of intellectual engagement, but also because Louis was always generous with his time, a good listener and very supportive of his editorial board. Louis was passionate about the area of science represented by *Evolutionary Applications*, and his enthusiasm for building cross‐domain links between fundamental evolutionary research and application held strong over the many years of his excellent leadership. In recent years, Louis helped to champion efforts to find support for improving journal approaches to ensuring data and code quality and accessibility, something that he felt was very important. He was a wonderful scientist and colleague, and the field will be poorer without him.

**Maren Wellenreuther, Plant and Food Research and the University of Auckland, New Zealand**

Louis introduced me to *Evolutionary Applications*, and after one of our many vigorous discussions that stretched science, politics and society, he suggested that we compile a Special Issue on ‘Women's contribution to basic and applied evolutionary biology’ in 2016. At that time, I was active in the STEM network for *women in science* at the University of Lund in Sweden, and we had many discussions about the need to highlight the accomplishments of women in science and to let these shine so that others can see them and be inspired. It was the first time that I was the guest editor for a Special Issue, and he supported me throughout the process and gently provided constructive feedback and encouragement (Wellenreuther & Otto, 2016). It was shortly after that he invited me to become an Associate Editor for *Evolutionary Applications* and I have thankfully accepted that role. For me, Louis has been an inspiring role model who was able to walk the golden line between excellent fundamental and applied research. Louis' legacy will endure for years to come because he successfully translated his discoveries into tangible impacts on people and society where it truly made a difference. In my view, this represents the pinnacle of scientific achievement—not merely filling knowledge gaps, but conducting science with the purpose of enhancing our coexistence and harmony with one another and the environment.

Though Louis took immense pride in his accomplishments, he at the same time remained remarkably humble. He once confided in me, acknowledging the ephemeral nature of time, that he thought that much of his scientific work might eventually fade from the memory of people. Nevertheless, he said, with unwavering determination in his voice, ‘I aspire to be remembered as a person of integrity and kindness’.

**José Manuel Yáñez, Facultad de Ciencias Veterinarias y Pecuarias, Universidad de Chile, Chile**

Louis has also played a pivotal role in the relatively recent expansion of the coverage of *Evolutionary Applications* into quantitative genetics and genomics applied to animal breeding. His vision was crucial for the inclusion of topics aiming at deciphering the genetic underpinnings of complex traits, integrating genomic data into genetic improvement programs and investigating the consequences of domestication and artificial selection in a variety of animal species. By fostering a platform for cutting‐edge research in these areas, Louis has contributed significantly to the journal's mission of bridging the gap between evolutionary biology and practical applications, thus advancing our understanding of how genomics can be harnessed for the sustainable improvement of terrestrial and aquatic organisms.

**Jiasui Zhan, Swedish University of Agricultural Sciences, Sweden**

Louis was a long‐time editor‐in‐chief of *Evolutionary Applications*, guiding and promoting the sustainable development of the journal. He was not only an outstanding scientist and a dedicated editor but also an approachable advisor. Before taking my role as Associate Editor for the journal, I contacted Louis multiple times with questions about review decisions and processes. He was always patient and willing to listen and answered my inquiries promptly and professionally. In the days before his death, he was still thinking about organizing a special issue for the journal.

**Xiangjiang Zhan, Key Laboratory of Animal Ecology and Conservation Biology, Institute of Zoology, Chinese Academy of Sciences, China**

I have known Louis since I studied in the UK. It was him who invited me to join the editorial team. He was always humorous and helpful. His work has illustrated many aspects of animal conservation and management, locally and globally.

Louis' passion for science, fish and the people they support was palpable. Louis' scientific spirit lives on in the graduate students, postdocs, research professionals and research assistants that he mentored over the years and collaborators that he interacted with. Louis inspired many people wherever he went. We will miss him dearly.

## ACKNOWLEDGMENTS

The authors would like to acknowledge all people that have known Louis, or that have crossed paths with his work. This tribute is to celebrate the impact that he has left behind.

## CONFLICT OF INTEREST STATEMENT

The authors have no conflict of interest.
